# A Review of Bacterial Colonization on Dental Implants With Various Hygiene Instruments

**DOI:** 10.7759/cureus.47483

**Published:** 2023-10-22

**Authors:** Ashmita Chen, Hareem Ghaffar, Haslina Taib, Akram Hassan

**Affiliations:** 1 School of Dental Sciences, Universiti Sains Malaysia, Kelantan, MYS

**Keywords:** biofilm formation, peri-implant diseases, dental implant, hygiene instruments, bacterial colonization

## Abstract

Peri-implant diseases can still develop despite oral hygiene practices being maintained. Consequently, regular debridement must be carried out to ensure the implant is sustained. This review evaluated bacterial colonization on implants following the use of different hygiene instruments. A literature search was conducted in PubMed, ScienceDirect, and Scopus databases for articles published from 2012 to 2022. A total of 19 full-text papers were selected. The number of bacteria colonized was most commonly evaluated with a scanning electron microscope (SEM) or by colony-forming unit (CFU) counts, crystal violet assays, plaque index, probing depth, bleeding on probing, turbidity test, and live-dead assays. Rubber cup polishing with an abrasive paste showed a significantly greater reduction in biofilm formation compared with air abrasion with glycine powder, while the air abrasion treatment was found to be more efficient than piezoelectric, carbon, and stainless steel scalers. Surface treatment with Er, Cr: YSGG laser, and Er: YAG laser resulted in statistically significant superior dental biofilm removal compared with titanium curettes and photodynamic therapy. Air abrasion, plastic curette, titanium curette, and ultrasonic scaler showed no significant differences in bacterial colonization, but air abrasion and plastic curette were safer for zirconia implant decontamination. Furthermore, the titanium brush showed better results in decontaminating the implant surface than the Er: YAG laser. Although no single instrument or method could be considered as offering a gold standard in treating peri-implant diseases, the use of air abrasion with glycine powder, laser therapies, rubber cup polishing with an abrasive paste, and a titanium brush had high levels of cleaning efficacy and acceptance by patients.

## Introduction and background

Among all the innovations in dental materials and their successful applications, dental implants may be a great case of the coordinate’s framework of innovation and science [1]. Although dental implants can replace missing teeth, they can also lead to peri-implant diseases, such as peri-implantitis and peri-implant mucositis, resulting in implant failure [2]. Peri-implant disease is an aggregate term for reversible peri-implant mucositis and irreversible peri-implantitis [3].

Despite following appropriate oral hygiene practices, patients might still develop peri-implant diseases due to the hard-to-reach area of the abutments, such as the implant neck [4]. Regular debridement must be carried out by dental practitioners to ensure that the dental implant is sustained [5]. Ideally, the hygiene instruments used on the dental implant should efficiently and effectively remove plaque accumulation, while causing no damage to the implant surface [5]. However, some instruments can increase the roughness of the implant surface, which may eventually increase bacterial colonization of the surface and lead to peri-implant diseases [6].

This review aimed to evaluate, based on the existing literature, the bacterial colonization on dental implants following different hygiene instrumentation. This knowledge will provide dental practitioners with a guide on choosing the type of instruments that can limit damage and effectively remove plaque. Application of this knowledge could decrease the prevalence of peri-implant diseases and implant failures.

## Review

Materials and methods

Study Design and Search Strategy

This review is based on a narrative overview of previous research. Articles were retrieved from searches of computerized databases and authoritative texts and hand-searches of the literature. Searches were conducted using the following keywords: “bacterial colonization,” “different hygiene instruments,” “dental implant,” “biofilm formation,” “dental maintenance care,” and “peri-implantitis.” These keywords were used to identify articles and journals for review in this study.

Criteria for Inclusion and Exclusion

This study included articles with keywords of interest, were published in English, and were available from trusted data sources such as PubMed, ScienceDirect, and Scopus databases for articles published from 2012 to 2022 that met all the eligibility criteria. Studies that were published on Wikipedia or were from unknown sources, those published in languages other than English, and those for which the full text was unavailable were excluded.

Results

PubMed, ScienceDirect, and Scopus searches identified 204, 189, and 152 papers, respectively. The initial screening of titles and abstracts identified 19 full-text papers, all subsequently processed for data analysis. Fourteen of the 19 papers described in vitro studies, while the other five papers were in vivo [7-11]. Three of the in vivo studies were randomized controlled trials [8-10], one was a clinical trial [11], and another one was a pilot randomized clinical trial [7]. Menini et al. [8] conducted a randomized controlled trial that involved 85 patients (46 males and 39 females) aged 45 to 88 years, while the other previous study that was carried out by Menini et al. [9] included 30 patients (18 males and 12 females) aged 47 to 86 years. Another randomized controlled trial by Schmidt et al. [10] was conducted with eight patients (six females and two males) aged 53 to 75 years. Clinical trials conducted by Zielbolz et al. [11] involved 62 patients (35 males and 27 females) at seven practices, with a mean age of 55.21 ± 11.3 years. Twenty adult patients (seven males and 13 females) with an age range of 25 to 70 years were involved in the pilot randomized clinical trial [7]. Among the 14 in vitro studies, 12 used titanium implants or discs, one used zirconia implants, and one [12] used both zirconia and titanium discs.

For nine of the 19 studies, bacterial colonization after the instrumentation was assessed using a scanning electron microscope (SEM). In the other studies, colonization was evaluated by colony-forming unit (CFU) count, crystal violet assays or staining, plaque index, peri-implant probing depth, bleeding on probing, turbidity test, live-dead assays, atomic force microscopy, peri-implant spontaneous bleeding, DNA-DNA hybridization, optical microscopy, peri-implant probing depth, mucosal recession, modified gingival bleeding index, plaque control record, clinical attachment level, gingival margin, matrix metalloproteinase-8, and peri-implant crevicular fluid. The hygiene instruments mostly tested were titanium, plastic, carbon, and/or stainless-steel curettes; air polishing with glycine powder; air polishing with sodium bicarbonate; ultrasonic scaler with plastic and/or stainless-steel tips; Er; YAG laser; Er, Cr: YSGG and/or diode laser; rubber cup polishing; chlorhexidine; titanium brush; and photodynamic therapy. Cold atmospheric plasma, 40% citric acid, and implantoplasty were occasionally tested.

The titanium periodontal curette is a hygiene instrument that is commonly used for the debridement of implants. Two in vitro studies revealed no significant differences between surfaces debrided with a titanium curette instrument and a control surface [13,14]. In a clinical study, Schmidt et al. [10] found no statistically significant differences for any parameter, except for a significant difference in full-mouth plaque control between titanium curette and stainless-steel ultrasonic tip at 12 months (p=0.0018). Similarly, Al Ghazal et al. [7] conducted a study that showed no significant difference in bleeding on probing; however, there was a gradual reduction in probing depth by 0.8 mm over the 12-month period, but it did not reach statistical significance (p=0.35). In contrast, Eick et al. and Larsen et al. supported using titanium curette as there was a significant reduction of bacteria observed following instrumentation [15,16]. One of the studies comparing titanium and zirconia samples showed the highest optical density for a bacterial suspension in the titanium curette-treated group (p<0.05) on the titanium surface, but there were no significant changes in the zirconia surface [12].

The instrument that was experimented with the most was air polishing with glycine powder. No significant difference was found in several studies [7,11-14,17]. Meanwhile, other studies showed positive results for air polishing with glycine powder. Menini et al. [8] stated that glycine powder air polishing was the second most effective method in removing biofilm, achieving 74.5% removal and resulting in a statistically significant plaque reduction. Idlibi et al. and Tran et al. supported using air polishing with glycine powder as the instrument demonstrated a statistically significant reduction in biofilm removal [18,19]. Eick et al. ranked air polishing with sodium bicarbonate as the third most effective instrument in their study, and Ziebolz et al. stated that the instrument showed remarkable cleaning effectiveness based on a significant reduction in plaque index on treated surfaces [11,15]. For decontamination using ultrasonic scalers, there were no statistically significant differences found in the result of using plastic tips compared with the control (untreated) surface [8,13,20].

A significant reduction in bacterial colonization was observed on the surfaces treated with rubber cup polishing [17,21,22]. Di Salle et al. [17] stated that a significant (p<0.01) reduction in biofilm formation was only found for the rubber cup polishing of the Detartrine ZTM (DZ)-treated implant surface, with the treatment reducing biofilm formation by about 40% for both *Pseudomonas aeruginosa* and *Staphylococcus aureus*. Studies by Shahbudin et al. also showed a significant difference in the median value of log10 CFU where rubber cup polishing had the lowest value and *Streptococcus mutans* colonies were lowest on the surfaces treated by rubber cups [21,22]. However, a clinical trial by Schmidt et al. [10] showed that participants exhibited minimal signs of periodontal inflammation with statistically significant pocket depth improvement, but there were no statistically significant differences (p>0.05) for any parameter at baseline or 12 months.

Five studies involved testing lasers for instrumentation, and four of these used Er: YAG laser and demonstrated a significant reduction in biofilm removal [15,16,23,24]. Eick et al. [15] stated that the lowest values of remaining bacteria (reduction by 4.45 log10) were seen on the Er: YAG-treated surface. Similarly, Alagl et al. [24] also stated that surfaces treated with Er: YAG showed significantly fewer viable cells, and most of *Acinetobacter baumannii* and *Pseudomonas aeruginosa* were ablated from the treated surface. Larsen et al. [16] showed a significant reduction in the number of attached *Porphyromonas gingivalis* (p<0.001). Er: YAG laser-treated surfaces had the lowest live-to-dead bacterial ratio in the study conducted by Al-Hashedi et al. [23]. The remaining laser study used a diode laser for decontamination and showed a decrease in the amount of biofilm [18].

Summaries of studies evaluating the decontamination of implant surfaces with different hygiene instruments and the most appropriate hygiene instrument used to decontaminate implant surfaces are presented in Tables 1-2, respectively.

**Table 1:** Summary of studies evaluating the decontamination of implant surfaces with different hygiene instruments AFM: atomic force microscopy; BOP: bleeding on probing; CAL: clinical attachment level; CAP: cold atmospheric plasma; CFU: colony-forming unit; DZ: Detartrine ZTM; Er, Cr: YSGG, erbium, chromium-doped yttrium, scandium, gallium, and garnet; Er: YAG, erbium-doped yttrium aluminum garnet; GBI: modified gingival (mucosal) bleeding index; GM: gingival margin; MMP-8: matrix metalloproteinase-8; MR: mucogingival recession; PCR: plaque control record; PD: probing depth; PI: plaque index; PPD: peri-implant probing depth; SB: peri-implant spontaneous bleeding; SEM: scanning electron microscope; SSC: stainless steel curettes.

References	Biofilm analysis	Results
[17]	AFM, CFU, and crystal violet staining	AFM showed the surface roughness increased after using the air-polishing device and ultrasonic scaler, while a significant reduction was noticed after using a curette or polishing with DZ abrasive paste. In addition, the DZ-treated implant surfaces had a significant reduction in biofilm formation.
[13]	SEM and CFU	Quantitative scoring revealed that SSC caused a significantly rougher surface than other instruments. After bacterial colonization, no significant differences were evident between control or instrumented surfaces in either culture.
[22]	CFU	The highest number of bacterial colonies was observed in the sample treated with air polishing, followed by the control and the abutment treated with a rubber cup with pumice powder (the lowest number of colonies).
[8]	PI, SB, PD, and BOP	Glycine air polishing following sponge floss application resulted in the greatest reduction of plaque accumulation around the implants.
[15]	CFU	Using Er: YAG resulted in significantly superior dental biofilm removal on titanium surfaces compared with the other treatments.
[19]	SEM and crystal violet assays	Glycine powder delivered through an air abrasion eliminated the most biofilm. Mechanical instruments were the least effective at eliminating biofilm across all surfaces and caused the greatest level of surface alterations.
[20]	SEM	After brushing with a dentifrice, the treated surfaces in all the treatment groups showed significantly fewer bacteria than the untreated surfaces (control group).
[21]	SEM	Rubber cups with pumice powder-treated implant surfaces resulted in lower amounts of *Streptococcus mutans* colonization than air-polishing instrumented surfaces.
[24]	SEM and CFU	The SEM analysis showed no significant change in the implant surface topography. A significant biofilm reduction was observed on the surface treated with Er, Cr: YSGG laser compared with chlorhexidine and diode laser-treated surfaces.
[16]	DNA-DNA hybridization	The titanium curette significantly altered the micro-texture of the implant surface. Neither the Er: YAG laser nor the chitosan brush significantly altered the surface.
[14]	SEM and turbidity test	No significant differences in bacterial adhesion were found between the different types of decontamination methods used.
[18]	Live-dead staining and SEM	Exposure to CAP significantly reduced the viability and quantity of biofilms compared with the positive control groups.
[25]	SEM and crystal violet assay	Re-colonization of *Streptococcus gordonii* after treatment was lowest after air abrasion, titanium brush, and implantoplasty versus control, but there was no difference between the plastic curette and the control treatment.
[12]	Optical microscope and turbidity test	Among the titanium samples, the titanium curette treatment group showed significantly higher optical density of bacteria colonization. As for the zirconia samples, the differences in bacterial colonization between groups were nonsignificant.
[23]	SEM and live-dead assays	Titanium brushes showed better results in decontaminating the implant surface than curettes and Er: YAG laser. Alternatively, Er: YAG laser-treated surfaces showed the lowest live-to-dead bacterial ratio.
[11]	PPD, MR, and BOP	For manual curettes, sonic-driven scaler, and prophylaxis brush, there were no significant implant-related differences between baseline and follow-up found in PPD, MR, and BOP. For prophylaxis brush showed a significant difference between baseline and follow-up in PPD. The type of implant, the location of the implant, and the age of the subject significantly affected BOP.
[9]	SB, PI, PD, and BOP	The PI reduction was significantly higher for glycine air polishing and bicarbonate air polishing than a carbon-fiber curette.
[10]	PD, BOP, GBI, PCR, CAL, GM, MMP-8, and periopathogens	Minimal signs of periodontal inflammation were exhibited with statistically significant PD improvement and overall CAL after 1 year. Implants showed no statistically significant differences between or within groups at baseline or 12 months for any parameter, except MMP-8 decreased significantly for stainless steel ultrasonic tip (PS), and after 12 months, PCR showed a significant difference between titanium curettes and PS.
[7]	BOP and peri-implant crevicular fluid analysis	No significant difference in BOP was found between the two treatment methods. Both debridement techniques showed a similar reduction of BOP. IL-6 was the only cytokine out of six investigated that correlated with a clinical parameter (BOP).

**Table 2:** Summary of studies identifying the most appropriate hygiene instrument used to decontaminate implant surfaces Er: YAG: erbium-doped yttrium aluminum garnet; Er, Cr: YSGG: erbium, chromium-doped yttrium, scandium, gallium and garnet; PEEK: polyether ether ketone; SLA: sandblasted, large grit, acid-etched; DZ: Detartrine ZTM; SCC: stainless steel curettes; PDT: photodynamic therapy; RC: rubber cup' AP: air polishing; TC: titanium curette; PC: plastic curette; US: ultrasonic scaler; UC: untreated control; GC: gas control; CAP: cold atmospheric plasma; AFP: air polishing powder

References	Hygiene Instrument tested	Conclusion
[17]	Air polishing device, ultrasonic scaler, curette, polishing with DZ abrasive paste	Both atomic force microscopy and antibiofilm analyses indicated that using DZ abrasive paste can be considered the prophylactic procedure of choice for managing peri-implant diseases and for therapy-resistant cases of periodontitis.
[13]	SCC, titanium curettes, air polishing with glycine-based (perio powder), air polishing with glycine-based (soft powder), air polishing with glycine-based (erythritol powder), ultrasonic with stainless steel, ultrasonic with plastic coated instruments	No significant differences were noted in the surface characteristics (except for SSC) or bacterial colonization based on one-time instrumentation.
[22]	Air polishing, rubber cup with pumice powder	Rubber cups with pumice powder showed lesser amounts of *Streptococcus mutans* colonization than air polishing treated and untreated surfaces due to surface alteration as it created a smoother surface topography.
[8]	Glycine air-polishing (G) and use of sponge floss vs dental sponge floss only, G vs ultrasonic device with a PEEK fiber tip-coating, G vs carbon fiber curette and sponge floss	Glycine air-polishing in professional oral hygiene of implant-supported restorations is highly effective and comfortable.
[15]	Gracey or titanium curettes, Er: YAG laser, PDT, titanium curettes with adjunctive PDT	Subgingival biofilm ablation and in particular decontamination of titanium implant surfaces with an Er: YAG laser seem to be a promising approach.
[19]	Air abrasion with glycine powders, air abrasion with sodium bicarbonate, air abrasion with calcium carbonate, piezoelectric scaler, carbon scaler, stainless steel scalers, chemical protocol using 40% citric acid	Both glycine powder in an air polisher and 40% citric acid application gave minimal alterations across all implant surfaces, with glycine the superior method in terms of biofilm removal.
[20]	Ultrasonic scalers with metal, ultrasonic scalers with plastic, and ultrasonic scalers with carbon	Titanium fixture surface treatment with ultrasonic metal, plastic, or carbon tip significantly enhanced bacterial removal efficacy of brushing.
[21]	RC with pumice powder, AP	The titanium surface topography after treatment with RC showed lower *Streptococcus mutans* colonization compared to AP samples due to the smoothening effect of the instruments.
[24]	Er, Cr: YSGG, diode laser, chlorhexidine	The Er, Cr: YSGG laser is an effective choice of treatment modality for the decontamination of dental implant surfaces without damaging the surface topography.
[16]	Er: YAG laser, chitosan brush, titanium curette	The Er: YAG laser, chitosan brush, and titanium curettes appear equally effective in minimizing the number of bacteria adhering to the micro-textured SLA implant surface, whereas the titanium curettes uniquely altered the implant surface structure.
[14]	TC, PC, air abrasive device, US with stainless steel tip	Stainless steel tips of TC and US should be used cautiously due to the deposition of metallic residue on the surface. Air abrasive devices and plastic curettes cause fewer surface alterations and are safer for zirconia implant decontamination.
[18]	UC, GC, CAP, diode laser, air-abrasion, chlorhexidine	No single method achieved complete biofilm destruction but CAP may provide effective support to established decontamination techniques for treatment of peri-implant diseases.
[25]	Plastic curette, air abrasion, titanium brush, implantoplasty	Air abrasion, titanium brush, and implantoplasty were more effective than the plastic curette at removing the *Streptococcus gordonii* biofilm and preventing recolonization.
[12]	Titanium curette, carbon fiber reinforced plastic curette, ultrasonic scaling with carbon fiber tip, air polishing with glycine powder	Zirconia was less susceptible to surface changes after tested cleaning procedures compared to titanium. Titanium curette should be used with caution on titanium abutments.
[23]	Metal curettes, plastic curettes, titanium brushes, Er: YAG laser	Titanium brushes were more effective than curettes (metal or plastic) and Er: YAG laser in decontaminating titanium implant surfaces, although none of these techniques could completely eliminate surface contamination. Er: YAG laser was more effective than curettes and titanium brushes in killing the biofilm bacteria.
[11]	Manual curettes, sonically driven scaler and prophylaxis brush, chlorhexidine varnish, air polishing with glycine powder	All strategies were effective in preventing peri-implant diseases. The supplemental application of chlorhexidine varnish had no significant additional benefit.
[9]	Glycine air polishing, sodium bicarbonate air polishing, manual scaling with carbon-fiber curette	Professional oral hygiene on implants using glycine air polishing revealed high levels of both cleaning efficacy and patient acceptance.
[10]	TC, stainless steel ultrasonic tip, erythritol air polishing, RC	All tested treatment modalities gave comparable clinical improvements.
[7]	Low abrasive AFP and TC	Both treatment methods were proven effective in reducing peri-implant diseases and preventing further disease progression.

Discussion

Biofilm management is essential for treating peri-implantitis and preventing early indicators of inflammation, consequently helping to prevent peri-implant diseases. Clinicians require recommendations for safe and efficient instruments to effectively, but conservatively, remove soft and hard deposits without affecting the implant surface or impinging on its biocompatibility [13].

Titanium Curette

Titanium curette showed effectiveness by reducing the amount of biofilm on treated surfaces [15,16]. Although no significant differences were observed in any of the parameters at baseline, the full-mouth Plaque Control Record showed significantly different results between titanium curette and stainless-steel ultrasonic tip after 12 months. However, this outcome could be due to suboptimal plaque removal by the patients in the study rather than to possible roughening [10]. Al Ghazal et al. [7] stated that titanium curette and air polishing showed a similar reduction of bleeding on probing, but there was no significant difference between the instruments. Eick et al. [15] reported that pairing titanium curette with additional therapy was more efficient than treating the implant surfaces with curette alone. Surfaces treated with titanium curette were found to have the highest density of adhered bacteria due to deep scratches and increased surface roughness. Thus, it was advised to use titanium curette with care [12].

Air Polishing Device With Glycine Powder

Most of the studies supported the use of air polishing with glycine powder since it showed significant differences in terms of the effective removal of bacteria on implant surfaces [8,18,19,25]. One study revealed that titanium disks treated with Perioflow demonstrated less *Streptococcus gordonii* adhesion than the untreated surface, probably due to the difference in surface profiles observed using SEM. The low bacterial adhesion on the surfaces treated with the air-powder abrasive system could be explained by the presence of deposits of glycine powder [25].

Air Polishing Device With Sodium Bicarbonate

Menini et al. showed that air polishing with a sodium bicarbonate prophylactic procedure provides remarkable cleaning effectiveness compared with manual debridement [9]. Meanwhile, another study showed varied effectiveness of sodium bicarbonate air polishing with different titanium surfaces. It was ranked as the third most effective for sandblasted, large grit, acid-etched (SLA) surfaces but was considered the least effective for both abraded and polished titanium surfaces [19].

Ultrasonic Scaler With Plastic Tip

Although two other studies showed no significant differences [8,13], Park et al. [20] found that brushing using ultrasonic plastic tips significantly enhanced the efficacy of bacterial removal by 0.8% ± 1.9% of bacteria when the number of bacteria in the no-treatment group was considered 100%.

Rubber Cup Polishing

A statistically significant reduction of *Staphylococcus aureus* biofilm was observed for rubber cup polishing with DZ abrasive paste compared with the use of an air-polishing device, highlighting that it may be considered the treatment of choice for the management of peri-implant lesions, as well as for therapy-resistant cases of periodontitis [17]. Another study also showed a statistically significant reduction of *Staphylococcus aureus* biofilm formation on the rubber cup-treated surface compared with air polishing treatment and control groups [22]. Two studies showed that rubber cup instrumentation reduced the amount of *Streptococcus mutans* colonization compared with air polishing [18,24]. Another study confirmed the use of rubber cup polishing where the parameters signified health for the tested group [10].

Titanium Brushes

Combining titanium brushes and laser therapy could be an effective protocol for managing peri-implantitis in dental clinics. This protocol would involve an initial cleaning of contaminated implant surfaces with titanium brushes to remove bacteria and organic contaminants, followed by Er: YAG laser treatment to disinfect the surfaces and eradicate the remaining bacteria [23].

Laser

Er: YAG laser: All the studies that used the Er: YAG laser showed a significant reduction in bacteria from the treated surfaces [15,16,23]. Eick et al. [15] stated that ablation of subgingival biofilms, particularly the decontamination of titanium implant surfaces, with Er: YAG laser seemed promising and worth further investigation. Another study did not show any of the ablation properties claimed for Er: YAG laser on bacteria, but bactericidal activity was confirmed based on the instrumentation yielding the lowest live-to-dead ratio [23]. Er: YAG laser was found to be uniquely effective in removing both strains of virulent and avirulent *Porphyromonas gingivalis*, although there were no statistically significant differences among the treated groups [16].

Er, Cr: YSGG laser: Er, Cr: YSGG laser was suggested in one of the studies as an effective technique for reducing bacteria contamination on implant surfaces because it showed fewer viable cells compared with other treatment groups and untreated groups. The study also showed that the Er, Cr: YSGG laser could ablate most of the *Acinetobacter baumannii* and *Pseudomonas aeruginosa* from the implant surface [24].

Limitation

One of the limitations of this review is that the selection process and the evaluation of biases in the published article or journal regarding the topic are unknown. In addition, it is difficult to predict whether excluding studies published in languages other than English could bias the conclusions of this review. Another limitation in this review is the validity of the outcome intraorally. Five studies carried out in vivo were included [7-11], and the others were in vitro studies. The outcome of the hygiene instruments intraorally was unpredictable because of many variables. In addition, the parameters of in vivo studies were difficult to compare across studies. Further, some studies in vitro contaminated and decontaminated the titanium implant surface, but there were differences between these processes in vitro and intraorally or in vivo. Clinical trials are needed to correlate these results with in vivo effects to apply these instruments in implant maintenance therapy.

Recommendations

Daily oral hygiene home care and regular preventive maintenance appointments are required for implants regardless of the instrumentation. Instrumentation alone is insufficient to completely remove bacteria biofilm. Thus, additional chemotherapeutic agents can be used [13]. Contrarily, the study showed no significant difference between the control group that received mechanical debridement (titanium curette) and the test group that received additional use of antiseptic therapy. Thus, the trial concluded that mechanical debridement alone is sufficient to reduce pocket depth values in sites diagnosed with peri-implant mucositis [7]. Further studies need to be conducted regarding the new and different types of hygiene instruments to develop standardized protocols for clinicians to use instruments accordingly. In addition, more clinical studies should be carried out, and the clinical impact of these findings should be clarified [26].

## Conclusions

No single instrument could be considered a gold standard in treating peri-implant diseases, but the use of metallic instruments should be avoided on titanium implant surfaces due to the risk of damage to the implant surface. Such damage could alter the surface topography and thus increase bacterial deposition and plaque accumulation. Bacterial infection causes inflammation around the implant and is one of the main etiological factors underlying implant failure. To stop or prevent the progression of the disease, it is crucial to remove plaque and calculus completely from the implant surface, using cost-effective instruments that are light, disposable, or easy to use and, most importantly, do not cause any damage to the implant surface. The use of air abrasion with glycine powder, laser therapies, rubber cup polishing with an abrasive paste, and titanium brush reveal high levels of both cleaning efficacy and acceptance by patients.
